# Engaging leadership and nurses’ mental health in German acute care hospitals: the mediating role of job resources

**DOI:** 10.1186/s12912-026-04953-w

**Published:** 2026-06-29

**Authors:** Joan Kleine, Dorothea Kohnen, Alessandro Campione, Reinhard Busse

**Affiliations:** 1 Management im Gesundheitswesen, Technische Universität Berlin, Straße des 17. Juni 135, 10623 Berlin, Germany; 2 School of Nursing and Midwifery, University College Cork, Cork, Ireland; 3 Faculty of Psychology and Educational Sciences, KU Leuven, Leuven, Belgium

**Keywords:** Engaging leadership, Job resources, Nurse well-being, Mental health, Burnout, Depression, Anxiety

## Abstract

**Background:**

Nurses in German acute care hospitals face high levels of work-related stress, including symptoms of burnout, depression, and anxiety. This study investigates whether engaging leadership is associated with burnout, depressive, and anxiety symptoms among nurses and whether these associations operate through job resources.

**Methods:**

A cross-sectional online survey was conducted among registered nurses working in acute care hospitals participating in the German branch of Magnet4Europe. Data collection took place between September and December 2023. Engaging leadership was measured with a validated twelve-item scale. Job resources included role clarity, skill use, autonomy, performance feedback and opportunities for development. Burnout symptoms were measured using the Burnout Assessment Tool. Depressive and anxiety symptoms were assessed with the PHQ-2 and GAD-2. The preregistered analysis used complete case analysis and structural equation modelling to test direct and indirect associations.

**Results:**

In total, 1,502 nurses from eighteen hospitals were included in the analysis. Engaging leadership was strongly associated with perceived job resources. Job resources were associated with lower burnout, depressive, and anxiety symptoms. Engaging leadership also showed small direct associations with burnout and depressive symptoms, but not with anxiety symptoms. Indirect effects were significant across all three models, suggesting that job resources may be an important mechanism linking engaging leadership to nurses’ mental health.

**Conclusions:**

Nurses who experience more engaging leadership also report more job resources and fewer symptoms of mental ill-health. These findings suggest that leadership practices which support meaning, competence, connection and autonomy may strengthen resilience and reduce psychological strain among nursing staff in Germany. Leadership development and resource strengthening may therefore represent important strategies for improving nurses’ well-being and retention in German hospital settings.

**Trial registration:**

Not applicable. This cross-sectional secondary analysis was conducted within the Magnet4Europe project, which is registered in the ISRCTN registry (ISRCTN10196901, registered on 10 April 2020).

**Supplementary Information:**

The online version contains supplementary material available at 10.1186/s12912-026-04953-w.

## Background

Across Europe, nurses face growing work-related stress driven by high job demands, persistent staffing shortages, and emotional strain. The COVID-19 pandemic further intensified these pressures by disrupting clinical work environments and amplifying emotional demands. In its aftermath, more than one in five nurses across several EU countries reported considering leaving the profession [1]. Recent EU-wide survey data confirm the extent of the mental health burden among healthcare workers: according to the 2025 World Health Organization (WHO) MeND-survey (Mental Health of Nurses and Doctors in the European Union, Iceland, and Norway), more than one third of nurses reported symptoms of anxiety or depression, and one in five met the threshold for burnout symptoms [2]. Such developments threaten not only individual well-being but also the stability and resilience of healthcare systems.

Germany is no exception to this trend. Nurses in German hospitals face particularly high physical and psychological demands [3], driven by demographic change [4] and structural challenges such as high work intensity, shift work, and unfavorable nurse-to-patient ratios [5, 6]. Although Germany has a comparatively high number of nurses per capita, each nurse is typically responsible for a significantly higher number of patients than their counterparts abroad [5, 6]. This imbalance increases workload strain, may erode job resources, and is associated with poorer quality of care and higher mental health risks [7].

These contextual pressures highlight the need to better understand modifiable organizational factors that may support nurses’ mental health in German hospital settings. However, there remains a lack of research on the mechanisms linking leadership behavior to symptoms of anxiety and depression among nurses in German hospitals. While previous studies have documented high levels of burnout, depressive, and anxiety symptoms among nurses [8–12], less is known about how organizational resources may explain associations between leadership and these different dimensions of mental ill-health. Mental health among nurses is not only an occupational health issue but central to the sustainability of nursing as a profession [2, 7].

This underlines the urgent need for organizational strategies to protect and promote nurses’ mental health. Among the modifiable factors, leadership has emerged as a critical determinant. Evidence shows that nurses’ perceptions of leadership behavior shape how they experience both stress and support at work, and are associated with mental well-being [13, 14]. Leaders therefore play a central role in either contributing to or mitigating mental health problems among nursing staff [15–17]. Although previous research has linked positive leadership to work outcomes such as satisfaction, performance, or turnover intention, fewer studies have examined health-related outcomes, particularly depressive and anxiety symptoms [18–20].

This study was designed to address this gap by examining the relationship between leadership and nurses’ mental health, focusing on the concept of Engaging Leadership (EL) [17].

## Conceptual model and hypotheses

To investigate how leadership may benefit nurses’ mental health, this study applies the concept of EL – a relationship-oriented leadership approach grounded in Self-Determination Theory (SDT) [21] – within the Job Demands–Resources (JD–R) Leadership Model [17]. SDT posits that well-being and motivation arise when three basic psychological needs are fulfilled: autonomy, competence, and relatedness [21]. Leaders who support these needs may foster motivation and psychological resources, which are particularly relevant in demanding settings such as hospitals.

Unlike broader positive leadership approaches such as transformational or servant leadership, EL emphasizes concrete and observable behaviors that support employees’ basic psychological needs [22]. Specifically, engaging leaders aim to motivate, empower, and retain employees by actively fostering psychological resources [17, 23]. EL is operationalized through four core behaviors: inspiring (providing meaning and vision), strengthening (building competence and confidence), connecting (enhancing team cohesion and interpersonal relationships), and empowering (promoting autonomy and responsibility) [17, 22]. In nursing, where staff often work under high pressure with limited autonomy and strong emotional demands, such behaviors may be particularly relevant for strengthening motivation and psychological well-being [15].

The JD–R Leadership Model [17, 24] provides a broader framework for understanding these mechanisms. It posits that leadership behaviors are associated with employee well-being partly by shaping job resources such as autonomy, feedback, team support, role clarity, skill use, and development opportunities, which may buffer the effects of high job demands such as workload and emotional strain. EL is therefore conceptualized as a resource-enabling leadership style that may strengthen access to job resources rather than directly reducing demands.

In this study, three dimensions of mental ill-health are examined. Burnout symptoms are conceptualized as a work-specific syndrome characterized by exhaustion, mental distance, and cognitive and emotional impairment [25]. Depressive symptoms refer to persistent low mood and loss of interest or pleasure, assessed as a general psychiatric symptom domain that extends beyond the work context [26]. Anxiety symptoms refer to persistent feelings of nervousness, worry, and difficulty controlling anxious thoughts [27]. Because burnout, depressive, and anxiety symptoms are linked not only to high demands but also to insufficient resources [24, 28], job resources may represent an important mechanism linking EL to nurses’ mental health.

Prior studies suggest that EL is associated with higher work engagement and lower emotional exhaustion, especially in resourceful work environments [13, 15]. However, it remains unclear whether these associations extend beyond burnout-related outcomes to other dimensions of mental ill-health, such as depressive and anxiety symptoms, and whether these associations are statistically mediated by perceived job resources. This is particularly relevant in the German hospital context, where nursing remains characterized by comparatively high workload pressures, limited autonomy, pronounced hierarchical structures, and slower academic professionalization than in many other European countries [6, 29, 30]. These systemic features make Germany a relevant setting for examining whether leadership behaviors that strengthen job resources are associated with nurses’ mental health under demanding conditions.

Data for this analysis are drawn from the third survey wave of the German branch of Magnet4Europe, a Horizon 2020-funded project focusing on improving nurse well-being and care quality in European hospitals by adapting the Magnet Model, which was originally developed in the United States [31]. A country-specific analysis was chosen because empirical evidence on the mechanisms linking leadership, job resources, and different dimensions of mental ill-health within German hospitals remains scarce.

To address these gaps, we examine whether and to what extent EL is associated with nurses’ mental health in 18 German acute care hospitals. Specifically, we test whether associations between perceived EL and burnout, depressive, and anxiety symptoms are mediated by nurses’ perceived access to job resources. Based on the JD–R Leadership Model and SDT, we test the following hypotheses:

### **H1**

Perceived engaging leadership is positively associated with perceived job resources among nurses.

### **H2a–c**

Perceived job resources are negatively associated with indicators of mental ill-health, namely (a) burnout symptoms, (b) depressive symptoms, and (c) anxiety symptoms.

### **H3a–c**

Perceived job resources mediate the relationship between perceived engaging leadership and (a) burnout symptoms, (b) depressive symptoms, and (c) anxiety symptoms.

### **H4a–c**

Perceived engaging leadership is directly negatively associated with (a) burnout symptoms, (b) depressive symptoms, and (c) anxiety symptoms.

## Method

### Study design and setting

We conducted a cross-sectional survey between September and December 2023 in acute care hospitals participating in the German branch of Magnet4Europe [31]. Although data collection occurred within the broader Magnet4Europe project, the present study does not evaluate intervention effects. Participating hospitals were engaged in organizational development activities aimed at improving work environments and leadership culture as part of the Magnet4Europe initiative. Thus, findings may reflect relationships observed in hospitals that had already begun strengthening supportive leadership practices. Ethical approval for the study was granted by the Ethics Committee of Charité – Universitätsmedizin Berlin (EA1/243/20).

### Recruitment

Eligible participants were registered nurses with an EU-recognized nursing qualification working in patient care, including intensive care and emergency departments, at one of the 18 participating hospitals. Recruitment was coordinated by designated project coordinators at each site, who distributed hospital-specific survey links via email to nursing staff. Additional strategies such as flyers, posters, and in-person outreach were used to increase participation. In total, 9,940 nurses were invited to participate between September and December 2023. Descriptive characteristics of respondents are reported in the Results section (Table 1).

**Table 1:** Sample characteristics (*N* = 1,502)

Variable	Mean ± SD (Median, IQR)
Age (years)	40.9 ± 11.7 (40; 31–51)
Work experience (years)	
– Total	18.6 ± 11.9 (18; 8–28)
– In current hospital	14.9 ± 11.3 (12; 5–24)
	**n (%)**
**Gender**	
– Female	1,155 (76.9%)
– Male	341 (22.7%)
– Diverse	6 (0.4%)
**Position**	
– Staff Nurse	1,145 (76.2%)
– Nurse Manager	325 (21.6%)
– Advanced Practice Nurse	32 (2.1%)
**Educational attainment**	
– Bachelor’s degree in nursing	222 (14.8%)
– Master’s degree in nursing	47 (3.1%)

Note. SD = standard deviation; IQR = interquartile range. Percentages may not total 100% due to rounding

### Measures

#### Engaging leadership (predictor)

Perceived EL was assessed with the 12-item Engaging Leadership Scale [17, 22], covering four subdimensions (inspiring, strengthening, connecting, empowering; 3 items each). Consistent with previous research using the Engaging Leadership Scale as an overall measure [15, 17, 22], the scale was used as a total score. Items were rated on a five-point Likert scale (1 = strongly disagree to 5 = strongly agree). A German translation was used. In the German version of the survey, the term ‘Vorgesetzter’ was used to refer to ‘supervisor.’ Example items were: “My supervisor encourages team members to develop their talents as much as possible.” (strengthening), “My supervisor promotes team spirit.” (connecting), “My supervisor encourages team members to voice their opinions” (empowering), and “My supervisor is inspiring” (inspiring). Internal consistency for the total scale was excellent (Cronbach’s α = 0.97).

#### Job resources (mediator)

In accordance with the preregistered analysis plan [32], job resources were measured with 11 items from the Energy Compass [33], covering job autonomy (4 items), role clarity (2 items), performance feedback (3 items), skill use (1 item), and opportunities for growth and development (1 item). This approach was chosen to capture the broader resource-enabling pathway proposed by the JD–R Leadership Model rather than resource-specific associations. Items were rated on a five-point Likert scale (1 = never to 5 = always). An example item was “Does your supervisor provide information about how well you perform your job?” (performance feedback) or “Can you decide when you perform your work?” (job autonomy). The included resources reflect conceptually related aspects of the psychosocial work environment, and the composite demonstrated satisfactory internal consistency (Cronbach’s α = 0.86).

#### Mental ill-health outcomes (dependent variables)

**Burnout symptoms** were assessed with the short version of the Burnout Assessment Tool (BAT; 12 items across four dimensions: exhaustion, mental distance, emotional impairment, cognitive impairment; Cronbach’s α = 0.90) [25, 34]. Example items include “At work, I feel mentally exhausted” (exhaustion), “I feel a strong aversion towards my job” (mental distance), “At work, I may overreact unintentionally” (emotional impairment), and “When I’m working, I have trouble concentrating” (cognitive impairment). For descriptive purposes, BAT-12 mean scores were additionally classified using published pooled European BAT-12 cut-off values [35, 36]. Scores of 2.54–2.95 were classified as indicating risk for burnout symptoms, and scores ≥ 2.96 as indicating very high risk.

**Depressive symptoms** were measured with the Patient Health Questionnaire-2 (PHQ-2; 2 items) [26]. Respondents were asked to indicate how often they had been bothered by depressive symptoms over the past two weeks (e.g., “Feeling down, depressed or hopeless”) on a 4-point scale ranging from 0 (not at all) to 3 (nearly every day). Scores range from 0 to 6, with higher scores indicating more severe depressive symptoms (inter-item correlation *r* = 0.65).

**Anxiety symptoms** were measured with the Generalized Anxiety Disorder Scale-2 (GAD-2; 2 items) [27]. Respondents were asked to indicate how often they had been bothered by anxiety symptoms over the past two weeks (e.g., “Feeling nervous, anxious or on edge”) on a 4-point scale ranging from 0 (not at all) to 3 (nearly every day). Scores range from 0 to 6 (inter-item correlation *r* = 0.66).

#### Covariates

Covariates included age (continuous), gender (female vs. other), educational level (bachelor’s degree in nursing vs. no bachelor’s degree in nursing; master’s degree in nursing vs. no master’s degree in nursing), and professional position (leadership position or advanced practice nurse vs. staff nurse).

### Statistical analysis

Analyses were conducted in R (version 4.3.2.) using the *lavaan* package (v0.6–16) [37]. Descriptive statistics and Pearson correlations were computed for all study variables. Skewness and kurtosis values were within acceptable limits (absolute skewness < 2; absolute kurtosis < 7), indicating approximate normal distributions [38]. Reliability was assessed using Cronbach’s α or inter-item correlations.

Structural equation modeling (SEM) was performed to test three separate models, each with one outcome (burnout symptoms, depressive symptoms, anxiety symptoms). EL was modeled as a second-order latent variable with four first-order factors. Job Resources was included as a composite observed variable, and outcomes were modeled as observed variables. Model fit was evaluated using the Comparative Fit Index (CFI) and Tucker–Lewis Index (TLI). Values above 0.90 indicated an adequate fit, while values above 0.95 suggested an even better fit. Additionally, the root mean squared error of approximation (RMSEA) and the standardized root mean residual (SRMR) were considered, with recommended thresholds of <0.06 (RMSEA) and <0.08 (SRMR) [39, 40]. Indirect effects were estimated with bootstrapping (5,000 resamples, 95% CI). Analyses included all covariates. As the preregistered research question focused on individual-level associations, models were estimated at the individual level. Complete case analysis was used because the proportion of incomplete questionnaires was low: 1,502 of 1,569 respondents completed the questionnaire in full (95.7%). Multiple imputation was therefore not applied.

#### Pre-registration

The analysis plan, including hypotheses and statistical procedures, was preregistered on the Open Science Framework prior to conducting inferential analyses (OSF registration: 10.17605/OSF.IO/S549E) [32].

## Results

### Sample characteristics

Of 9,940 invited nurses, 1,569 responded (response rate: 15.8%), and 1,502 completed the questionnaire in full (completion rate: 95.7%). The final analytic sample thus comprised 1,502 nurses from 18 German acute care hospitals.

Table 1 presents the demographic and professional characteristics of the final sample (*N* = 1,502). The majority of participants were female (76.9%). The mean age of the total sample was 40.9 years (SD = 11.7). On average, nurses reported 18.6 years of total work experience and 14.9 years in their current hospital. Most participants were staff nurses (76.2%). Full sample characteristics are presented in Table 1.

### Descriptive statistics, correlations, and prevalence of mental ill-health symptoms

Table 2 presents descriptive statistics, reliability coefficients, and intercorrelations among the main study variables. All measures demonstrated satisfactory internal consistency (Cronbach’s α = 0.86–0.97).

**Table 2:** Reliability coefficients and bivariate correlations (Pearson r) among main study variables (*N* = 1,502)

Variable	k	M	SD	α / r	1	2	3	4	5
1. EL	12	3.34	0.91	α = 0.97	1.00				
2. JR	11	3.44	0.58	α = 0.86	0.63***	1.00			
3. Burnout (BAT-12)	12	2.29	0.62	α = 0.90	−0.37***	−0.49***	1.00		
4. Depression (PHQ-2)	2	1.47	1.39	r = 0.65	−0.30***	−0.39***	0.71***	1.00	
5. Anxiety (GAD-2)	2	1.32	1.39	r = 0.66	−0.19***	−0.28***	0.62***	0.65***	1.00

Note. k = number of items; M = mean; SD = standard deviation; α = Cronbach’s alpha; r = Pearson correlation coefficient. ***p < 0.001. EL = Engaging Leadership; JR = Job Resources; BAT-12 = 12-item Burnout Assessment Tool; PHQ-2 = Patient Health Questionnaire-2; GAD-2 = Generalized Anxiety Disorder Scale-2. PHQ-2 and GAD-2 values represent mean of summed scores (score range 0–6)

EL was positively associated with job resources (*r* = 0.63, *p* < 0.001) and negatively related to burnout symptoms (*r* = −0.37, *p* < 0.001), depressive symptoms (*r* = −0.30, *p* < 0.001), and anxiety symptoms (*r* = −0.19, *p* < 0.001). Similarly, job resources showed significant negative correlations with all three mental ill-health outcomes (*r* = −0.49 to −0.28, all *p* < 0.001).

Table 3 presents descriptive classifications of elevated mental ill-health symptoms based on cut-off values. Using published pooled European BAT-12 cut-off values [35, 36], 279 nurses (18.6%) were classified as being at risk for burnout symptoms and 198 nurses (13.2%) as being at very high risk. Overall, 477 nurses (31.8%) were classified as being at risk or at very high risk for burnout symptoms. Based on the established PHQ-2 and GAD-2 cut-off score of ≥ 3 [26, 27], 16.2% of nurses reported clinically relevant depressive symptoms and 15.2% reported clinically relevant anxiety symptoms.

**Table 3:** Descriptive classification of elevated mental ill-health symptoms based on cut-off values (*N* = 1,502)

Measure	Classification / cut-off	n	%
Burnout (BAT-12)	At risk: 2.54–2.95	279	18.6%
Burnout (BAT-12)	Very high risk: ≥2.96	198	13.2%
Depression (PHQ-2)	≥3	244	16.2%
Anxiety (GAD-2)	≥3	229	15.2%

Note. BAT-12 = 12-item Burnout Assessment Tool; PHQ-2 = Patient Health Questionnaire-2; GAD-2 = Generalized Anxiety Disorder Scale-2. BAT-12 classifications are based on established cut-off values: at risk = 2.54–2.95 and very high risk ≥ 2.96 [35, 36]. These classifications indicate risk levels for burnout symptoms and should not be interpreted as clinical burnout diagnoses. Scores on the PHQ-2 and GAD-2 range from 0 to 6; a cut-off score of ≥ 3 indicates clinically relevant symptoms of depression or anxiety, respectively [26, 27]

### Structural equation modeling findings

Three SEMs were estimated to test whether the relationship between EL and nurses’ mental ill-health outcomes was mediated by job resources. Across all models, EL behavior was positively associated with job resources (β = 0.64, *p* < 0.001). In turn, job resources were negatively related to burnout symptoms (β = −0.42, *p* < 0.001), depressive symptoms (β = −0.33, *p* < 0.001), and anxiety symptoms (β = −0.27, *p* < 0.001).

Direct effects of EL on nurse mental ill-health outcomes were significant for burnout symptoms (β = −0.11, *p* < 0.001) and depressive symptoms (β = −0.10, *p* < 0.01), but not for anxiety symptoms (β = −0.02, ns). Indirect effects via job resources were significant across all models (burnout: β = −0.27, depression: β = −0.21, anxiety: β = −0.17; all *p* < 0.001), suggesting that the association between EL and nurses’ mental ill-health may operate partly through perceived job resources.

Model fit indices indicated excellent fit in all three SEMs (Table 4).

**Table 4:** Model fit indices for the three SEMs (*N* = 1,502)

Outcome	CFI	TLI	RMSEA	SRMR	Fit^a^
Burnout (BAT)	0.983	0.979	0.047	0.018	***
Depression (PHQ-2)	0.982	0.978	0.048	0.018	***
Anxiety (GAD-2)	0.983	0.978	0.056	0.019	***

Note. CFI = Comparative Fit Index; TLI = Tucker–Lewis Index; RMSEA = Root Mean Square Error of Approximation; SRMR = Standardized Root Mean Square Residual

^a^ Fit evaluation based on recommended thresholds: *** = excellent fit (CFI/TLI ≥ 0.95, RMSEA < 0.06, SRMR < 0.05); ** = good fit (CFI/TLI ≥ 0.90, RMSEA < 0.08, SRMR < 0.08) (Hu & Bentler, 1999; Van de Schoot et al., 2012)

**Fig. 1:**
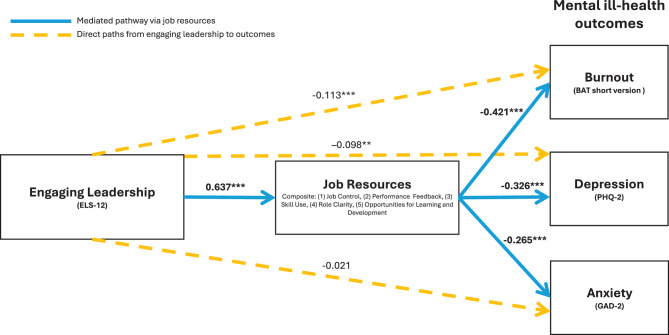
Structural equation model of the associations between engaging leadership, job resources, and mental ill-health outcomes among nurses (*N* = 1,502). Note. Standardized path coefficients (β) are shown. Blue solid paths indicate the mediated pathway via job resources, including the path from engaging leadership to job resources and the paths from job resources to each mental ill-health outcome. Yellow dashed paths indicate direct paths from engaging leadership to each mental ill-health outcome. EL = Engaging Leadership; ELS-12 = Engaging Leadership Scale (12-item version); JR = Job Resources; BAT = Burnout Assessment Tool (short version); PHQ-2 = Patient Health Questionnaire-2; GAD-2 = Generalized Anxiety Disorder Scale-2. ***p < 0.001, **p < 0.01, *p < 0.05

Figure 1 visualizes the tested structural equation model, showing standardized path coefficients for all direct and indirect associations. Figure 1 is available in the published version. The figure illustrates that EL was positively related to job resources, which in turn were negatively associated with burnout, depressive, and anxiety symptoms. Together, EL and job resources explained 25% of the variance in burnout symptoms, 16% in depressive symptoms, and 8% in anxiety symptoms (Table 5).

**Table 5:** Standardized path coefficients in the three SEMs

Path	Burnout (BAT) β	Depression (PHQ-2) β	Anxiety (GAD-2) β
EL → JR	0.64***	0.64***	0.64***
JR → Outcome	−0.42***	−0.33***	−0.27***
EL → Outcome (direct)	−0.11***	−0.10**	−0.02 (ns)
Indirect effect (EL → JR → Outcome)	−0.27***	−0.21***	−0.17***
Total effect	−0.38***	−0.31***	−0.19***
R^2^ JR	0.41	0.41	0.41
R^2^ Outcome	0.25	0.16	0.08

Note. EL = Engaging Leadership; JR = Job Resources; BAT = Burnout Assessment Tool; PHQ-2 = Patient Health Questionnaire-2; GAD-2 = Generalized Anxiety Disorder Scale-2. ***p < 0.001, **p < 0.01, ns = not significant. R^2^indicates the proportion of explained variance in the respective endogenous variable

Additional diagnostic and robustness analyses were conducted to ensure the stability of the findings. All models included covariates (age, gender, educational attainment, and leadership position), which did not substantively alter associations (Additional file 1: Table S1). Bootstrap analyses with 5,000 resamples confirmed the robustness of indirect and total effects (Additional file 2: Table S2). Variance Inflation Factors (VIFs) indicated no multicollinearity among predictors (all VIF < 2.5; Additional file 3: Table S3).

Overall, the results support the hypothesized mediation model, suggesting that the association between EL and nurses’ mental health may operate primarily through job resources.

## Discussion

This study examined how EL relates to nurses’ mental health in German acute care hospitals, focusing on the mediating role of job resources. Overall, the findings supported H1, as EL was positively associated with job resources, and H2a–c, as job resources were negatively associated with burnout, depressive, and anxiety symptoms. H3a–c were also supported, as indirect effects via job resources were significant for all three outcomes. Regarding direct associations, H4a and H4b were supported, as EL was directly and negatively associated with burnout and depressive symptoms. In contrast, H4c was not supported, as the direct association between EL and anxiety symptoms was not statistically significant. The non-significant direct association with anxiety symptoms may indicate that factors not captured in our model, such as individual vulnerability, immediate emotional states, family pressures, or other non-work-related stressors, play a larger role for this outcome. It may also suggest that the resource-related association between engaging leadership and anxiety symptoms is weaker or less direct than for burnout and depressive symptoms.

The findings suggest a consistent statistical mediation pattern through job resources, while also indicating that the direct association between EL and mental health may differ across outcome domains.

### Leadership, job resources, and mental health

The findings are consistent with and extend prior research suggesting that leadership behaviors are associated with the psychosocial work environment and nurses’ well-being [16, 20]. Consistent with SDT, EL includes behaviors that are theoretically assumed to support autonomy, competence, and relatedness; however, basic need fulfillment itself was not assessed in the present study [21, 22]. Within the JD–R framework, leadership is theorized to be linked to employee well-being partly through job resources rather than only through direct reductions in job demands [17, 24]. Our findings are consistent with this assumption, as nurses who perceived higher EL also reported greater access to job resources, which in turn was associated with fewer symptoms of mental ill-health. The substantial correlation between EL and job resources (*r* = 0.63) is consistent with previous research [15, 17] and may reflect the theoretically proposed link between engaging leadership and resourceful work environments. While these constructs are conceptually distinct - EL referring to specific leadership behaviors and job resources referring to perceived work environment characteristics - given the cross-sectional design, the directionality of this association cannot be established from the present data alone.

Job resources, in turn, were negatively associated with all three mental health outcomes, and indirect effects via job resources were significant across all models. However, the overall effect sizes were moderate. Together, EL and job resources explained 25% of the variance in burnout symptoms, 16% in depressive symptoms, and 8% in anxiety symptoms. Anxiety symptoms showed the lowest explained variance, suggesting that other individual, organizational, or non-work-related factors may play a larger role for this outcome. Overall, while leadership and job resources appear relevant to nurses’ mental health, substantial proportions of variance remain attributable to other individual, organizational, and contextual factors. Future studies could therefore examine whether specific job resources, such as autonomy, role clarity, feedback, skill use, or development opportunities, differ in their associations with engaging leadership and mental health outcomes. In hospital settings, these leadership behaviors are often enacted by nurse managers and ward leaders, who are closely involved in daily work processes, communication patterns, and access to resources. Developing EL competencies among nurse managers may represent a relevant and profession-specific approach to supporting nurses’ psychological well-being.

By including depressive and anxiety symptoms alongside burnout symptoms, this study broadens the evidence on leadership and mental health in nursing. Extending prior research on engagement and burnout [15], our results suggest that EL is also associated with depressive and anxiety symptoms, although the direct association with anxiety symptoms was not significant and explained variance was lowest for this outcome. These findings underscore the importance of conceptualizing leadership as part of the broader psychosocial work environment associated with psychological well-being, rather than as a direct determinant of distress.

### The German hospital context and Magnet4Europe

The study was conducted in German hospitals participating in Magnet4Europe, an initiative that aims to improve nurse well-being and work environments by promoting participatory and transformational leadership practices [31]. Although this study does not assess intervention effects, participating hospitals were engaged in organizational development activities aimed at improving leadership culture. These hospitals also self-selected into Magnet4Europe, indicating an explicit commitment to improving leadership, work environments, and well-being. As a result, levels of perceived EL and job resources in this sample may be somewhat higher than in hospitals without such commitment, which should be considered when generalizing the findings.

The German context provides a relevant setting for examining these dynamics. Compared with other European countries, nursing in Germany remains characterized by hierarchical structures, limited autonomy, and relatively slow academic professionalization [6, 30]. Relationship-oriented leadership behaviors (such as EL) that emphasize empowerment, competence, and connection may therefore be particularly relevant for nurses’ mental health in this system.

Our findings suggest that, even within hospitals actively engaged in leadership and work environment development through Magnet4Europe, nurses report substantial differences in perceived EL and available job resources. This may indicate that cultural change toward more supportive and participatory leadership may not occur uniformly across units. The observed associations are consistent with leadership behaviors promoted by Magnet principles, such as empowerment and shared decision-making, which align with shared governance approaches in nursing [41, 42].

### Strengths and limitations

Several limitations should be acknowledged. First, the cross-sectional design precludes causal inference. Although the proposed mediation model is theoretically grounded, reverse causation cannot be ruled out; nurses experiencing better mental health may also perceive their leaders and work environment more positively or may be better able to leverage available job resources effectively. In addition, job demands, such as workload, staffing pressure, and emotional labor, were not measured. We therefore could not examine potential demand–resource interactions, although the JD–R framework suggests that job resources may be particularly relevant under conditions of high job demands. Second, all measures were self-reported, raising potential for common-method bias. However, the use of the BAT instead of the Maslach Burnout Inventory (MBI) represents a methodological strength, offering a multidimensional and psychometrically robust assessment of burnout symptoms [25]. Furthermore, the three mental health indicators - burnout, depressive, and anxiety symptoms - showed substantial intercorrelations (*r* = 0.62–0.71), which may limit the extent to which truly differential conclusions can be drawn across outcomes. Although these constructs are conceptually distinct, their shared variance as indicators of general psychological distress should be considered when interpreting the differential findings reported in this study. Third, response bias may have affected the sample, as nurses experiencing the highest distress might have been less likely to participate, a potential survivor effect observed in similar surveys [43, 44]. Fourth, although nurses were recruited from 18 hospitals, the analysis focused on individual-level associations and did not model hospital-level random effects. All main variables were measured as individual perceptions or individual outcomes, and ward-level identifiers were not available, although leadership and job resources may be experienced most directly at the ward or unit level. Not modelling clustering may have led to underestimated standard errors if responses were correlated within hospitals or wards. Future studies should use multilevel designs with sufficient numbers of hospitals and ward-level identifiers to examine unit- and hospital-level variation in engaging leadership, job resources, and nurses’ mental health. Fifth, PHQ-2 and GAD-2 are brief screening instruments rather than diagnostic tools. Finally, the sample included hospitals participating in Magnet4Europe, which may not be representative of all German hospitals and could limit generalizability. A comparison with national data suggests that our sample is broadly representative in terms of age. The proportion of female nurses in our sample (76.9%) was somewhat lower than the national average of 84% reported for Germany [45], suggesting a slight overrepresentation of male nurses, which should be considered when interpreting the findings. Although gender was included as a covariate, the study was not designed to examine gender-specific mechanisms. Future studies could use gender-sensitive or gender-stratified designs to examine whether associations between engaging leadership, job resources, and mental health outcomes differ by gender.

The structural equation model applied in this study was theoretically sound and demonstrated excellent model fit, yet it represents a simplified framework. More complex causal structures, such as those modeled through directed acyclic graphs (DAGs), were not explored. As a result, potential collider bias or unobserved confounding cannot be fully ruled out [46, 47].

Despite these limitations, the study has several notable strengths. First, it draws on a large and diverse sample of more than 1,500 nurses from 18 acute care hospitals, providing robust statistical power and enhancing the reliability of estimates. Second, the analysis was preregistered on the Open Science Framework, which increases transparency, prevents data-driven hypothesis formulation, and enhances the credibility of the findings. Third, the study integrates EL, SDT, and the JD–R Leadership Model within one analytical framework, offering a coherent theoretical contribution to the understanding of leadership and nurse well-being. Fourth, by simultaneously considering burnout, depressive, and anxiety symptoms, the study extends previous research that has largely focused on single outcomes and shows consistent indirect associations across multiple dimensions of mental health. Finally, the excellent model fit and the robustness of findings across all sensitivity analyses support the methodological robustness of the results. 

## Implications

The findings show that nurses who perceive their leaders as more engaging report more job resources. Hospitals may use this insight to inform leadership practices that emphasize empowerment, shared decision-making, and supportive social interactions [48]. These behaviors align with Magnet principles and may contribute to work environments that feel more predictable, collegial, and professionally meaningful [49, 50]. Because EL-related behaviors may be developed through role modelling, coaching, and training [22, 51], hospitals may consider embedding them more systematically into leadership development and reward structures. In particular, nurse managers and clinical leaders may benefit from targeted development programs that promote EL behaviors and support their translation into everyday ward-level practice. Integrating EL competencies into structured nurse leadership frameworks and advanced nursing education may therefore be a promising approach to supporting workforce resilience and retention.

Job resources such as role clarity, constructive feedback, and opportunities for skill use were closely linked to lower burnout, depressive, and anxiety symptoms. Improving the availability and consistency of these resources, for example through clearer task allocation, reliable communication, and structured feedback routines, may therefore serve as a relevant approach to protect nurses’ well-being. This aligns with the JD–R Leadership Model, which conceptualizes job resources as aspects of work that support goal achievement, reduce strain, and foster motivation and well-being [33, 52]. Longer-term, these results highlight the potential value of nursing leadership and resource development for workforce retention and the sustainability of professional nursing practice.

## Conclusion

This study found that perceived EL was associated with more job resources and fewer symptoms of burnout, depression, and anxiety among nurses in German acute care hospitals. The findings suggest that job resources may represent a key mechanism in the association between leadership behavior and nurses’ psychological well-being, highlighting the relevance of resource-oriented leadership practices in demanding hospital settings.

## Electronic supplementary material

Below is the link to the electronic supplementary material.

Supplementary material 1

Supplementary material 2

Supplementary material 3

## Data Availability

The individual-level data underlying the findings of this study, after de-identification, are available from the corresponding author upon reasonable request and subject to approval by the Magnet4Europe consortium. Requests must include a methodologically sound research proposal.

## Abbreviations

BAT: Burnout Assessment Tool
CFI: Comparative Fit Index
DAG: Directed Acyclic Graph
EL: Engaging Leadership
GAD-2: Generalized Anxiety Disorder Scale–2
JD–R: Job Demands–Resources
JR: Job Resources
MBI: Maslach Burnout Inventory
OSF: Open Science Framework
PHQ-2: Patient Health Questionnaire–2
RMSEA: Root Mean Square Error of Approximation
SD: Standard Deviation
SDT: Self-Determination Theory
SEM: Structural Equation Modeling
SRMR: Standardized Root Mean Square Residual
TLI: Tucker–Lewis Index
VIF: Variance Inflation Factor
WHO: World Health Organization
