# Supplementary material for: Engaging leadership and nurses’ mental health in German acute care hospitals: the mediating role of job resources
Source: BMC Nurs. 2026 Jun 29;25:569. doi: 10.1186/s12912-026-04953-w (PMC13317338; doi:10.1186/s12912-026-04953-w)
Supplement: Supplementary file 1 — Supplementary material 1 [file 12912_2026_4953_MOESM1_ESM.docx]

Additional file 1

Table S1. Covariates predicting Job Resources, Burnout (BAT), Depression (PHQ-2), and Anxiety (GAD-2) in structural equation models (N = 1,502)

| Outcome | Covariate | Est. | SE | p | Std. β | Interpretation |
| --- | --- | --- | --- | --- | --- | --- |
| Job Resources | Age | 0.001 | 0.001 | .475 | 0.015 | n.s. |
|  | Female | 0.051 | 0.029 | .078 | 0.038 | n.s. |
|  | BSN degree | 0.011 | 0.036 | .763 | 0.007 | ns |
|  | MSN degree | 0.082 | 0.068 | .230 | 0.025 | ns |
|  | Leadership/APN | 0.138 | 0.030 | <.001 | 0.102 | Significant positive effect |
| Burnout (BAT) | Age | 0.000 | 0.001 | .760 | 0.007 | ns |
|  | Female | –0.009 | 0.034 | .798 | –0.006 | ns |
|  | BSN degree | 0.043 | 0.048 | .369 | 0.025 | n.s |
|  | MSN degree | 0.013 | 0.088 | .886 | 0.004 | n.s. |
|  | Leadership/APN | –0.091 | 0.035 | .009 | –0.063 | Significant protective effect |
| PHQ-2 | Age | –0.011 | 0.003 | <.001 | –0.095 | Older nurses report fewer depressive symptoms |
|  | Female | –0.116 | 0.080 | .146 | –0.035 | n.s. |
|  | BSN degree | –0.016 | 0.099 | .868 | –0.004 | n.s. |
|  | MSN degree | –0.171 | 0.181 | .345 | –0.022 | n.s. |
|  | Leadership/APN | –0.020 | 0.080 | .806 | –0.006 | n.s. |
| GAD-2 | Age | –0.010 | 0.003 | .001 | –0.086 | Older nurses report fewer anxiety symptoms |
|  | Female | 0.093 | 0.081 | .250 | 0.028 | n.s. |
|  | BSN degree | 0.087 | 0.104 | .399 | 0.022 | n.s. |
|  | MSN degree | –0.220 | 0.232 | .342 | –0.028 | n.s. |
|  | Leadership/APN | 0.047 | 0.086 | .582 | 0.015 | n.s. |

*Note. n.s. = not significant; BSN = Bachelor of Science in Nursing; MSN = Master of Science in Nursing; APN = Advanced Practice Nurse; BAT = Burnout Assessment Tool; PHQ-2 = Patient Health Questionnaire-2; GAD-2 = Generalized Anxiety Disorder Scale-2. Est. = unstandardized estimate; SE = standard error; Std. β = standardized coefficient.*
