# Supplementary material for: Engaging leadership and nurses’ mental health in German acute care hospitals: the mediating role of job resources
Source: BMC Nurs. 2026 Jun 29;25:569. doi: 10.1186/s12912-026-04953-w (PMC13317338; doi:10.1186/s12912-026-04953-w)
Supplement: Supplementary file 2 — Supplementary material 2 [file 12912_2026_4953_MOESM2_ESM.docx]

Additional file 2

Table S2. Bootstrap estimates for indirect and total effects (5,000 resamples)

| Outcome | Effect type | Est. | SE | z | p | 95% CI (percentile) | Std. β |
| --- | --- | --- | --- | --- | --- | --- | --- |
| Burnout (BAT) | Indirect (EL → JR → Burnout) | –0.169 | 0.016 | –10.81 | <.001 | [–0.201, –0.140] | –0.254 |
|  | Total (direct + indirect) | –0.240 | 0.019 | –12.76 | <.001 | [–0.277, –0.204] | –0.361 |
| PHQ-2 | Indirect (EL → JR → PHQ-2) | –0.292 | 0.034 | –8.62 | <.001 | [–0.359, –0.226] | –0.195 |
|  | Total (direct + indirect) | –0.432 | 0.043 | –9.96 | <.001 | [–0.516, –0.347] | –0.288 |
| GAD-2 | Indirect (EL → JR → GAD-2) | –0.243 | 0.035 | –6.85 | <.001 | [–0.314, –0.176] | –0.161 |
|  | Total (direct + indirect) | –0.275 | 0.045 | –6.06 | <.001 | [–0.367, –0.187] | –0.183 |

*Note. EL = Engaging Leadership; JR = Job Resources; BAT = Burnout Assessment Tool; PHQ-2 = Patient Health Questionnaire-2; GAD-2 = Generalized Anxiety Disorder Scale-2. Est. = unstandardized effect estimate; SE = standard error; z = z-value; CI = confidence interval; Std. β = standardized effect. Indirect effects represent the mediated paths from Engaging Leadership to the respective outcomes via Job Resources. Total effects represent the sum of direct and indirect effects. Confidence intervals are bias-corrected percentile bootstrap intervals based on 5,000 resamples. Negative coefficients indicate that higher Engaging Leadership is associated with lower levels of burnout, depressive symptoms, and anxiety via increased job resources. All reported effects were statistically significant at p < .001.*
