# Supplementary material for: Engaging leadership and nurses’ mental health in German acute care hospitals: the mediating role of job resources
Source: BMC Nurs. 2026 Jun 29;25:569. doi: 10.1186/s12912-026-04953-w (PMC13317338; doi:10.1186/s12912-026-04953-w)
Supplement: Supplementary file 3 — Supplementary material 3 [file 12912_2026_4953_MOESM3_ESM.docx]

Additional file 3

Table S3. Variance Inflation Factors (VIF) for all covariates

| Covariate | VIF |  |
| --- | --- | --- |
| Age | 1.05 |  |
| Female | 1.02 |  |
| BSN degree | 1.18 |  |
| MSN degree | 1.14 |  |
| Leadership/APN | 1.15 |  |
| Job Resources (mediator) | 1.07 |  |

Note. No indication of multicollinearity (all VIF < 2.5). BSN = Bachelor of Science in Nursing; MSN = Master of Science in Nursing; APN = Advanced Practice Nurse.
